# The business case for carbon farming in the USA

**DOI:** 10.1186/s13021-024-00253-5

**Published:** 2024-02-16

**Authors:** Alejandro Plastina, Haeun Jo, Oranuch Wongpiyabovorn

**Affiliations:** 1 Department of Economics, and Affiliated Faculty, Center for Agricultural and Rural Development, Iowa State University, 478E Heady Hall, 518 Farm House Ln, Ames, IA 50011-1054 USA; 2 Department of Economics, Iowa State University, 280A Heady Hall, 518 Farm House Ln, Ames, IA 50011-1054 USA; 3 Ness School of Management and Economics, South Dakota State University, Harding Hall 205, Brookings, SD, 57007 USA

**Keywords:** Cover crops, No-till, Carbon sequestration, Conservation practices, Net returns, Payments per output, Payments per practice, Agricultural carbon credits

## Abstract

U.S. agricultural producers are increasingly able to participate in private voluntary carbon initiatives that compensate their efforts to sequester CO_2_, reduce GHG emissions, and provide ecosystem services through eligible conservation practices. This study examines the potential effects of alternative private payment regimes (per practice vs. per output), prices paid to farmers relative to out-of-pocket costs (low vs. high), and the availability of information on CO_2_ sequestration (limited vs. full), on the adoption of cover crops and no-till in the United States, the resulting CO_2_ sequestration, and changes in farmers’ net returns. The analysis relies on a highly stylized model of heterogeneous farms calibrated with county-level agronomic data, and simulated for current estimates of GHG impacts of cover crop planting and no-till under different scenarios. Our results indicate that agricultural carbon markets can be profitable for U.S. farmers, although with substantial geographic variability, and that annual carbon sequestration could range between 17 and 75 million mtCO_2_e. Payments per output would incentivize higher carbon sequestration than payments per practice, but the former regime would be less favored by farmers as a unified group than the latter (due to lower aggregate net returns). However, if operators of farms with high carbon sequestration potential could decide the payment regime to be implemented, they would choose the payment per output regime (due to higher net returns per enrolled hectare). Total projected net changes in GHGs under payments per practice, based solely on county-average net GHG effects of cover crops and no-till, over-estimate actual total GHG sequestration (based on the entire distribution of net effects by county) by 2.1 and 14.2 million mtCO_2_e, or 18% and 21%, respectively.

## Background

While global agriculture and related land use emissions account for 17% of global greenhouse gas (GHG) emissions [5], the implementation of agricultural conservation practices that sequester atmospheric carbon dioxide (CO_2_) and store it as biomass and soil organic carbon (SOC) and reduce GHG emissions can substantially contribute to climate change mitigation [12]. As 98% of global cropland is potentially available for enhanced carbon sequestration through farming conservation practices [35], croplands have a strong potential to contribute to climate change mitigation and food security. Among the various conservation practices with the potential to mitigate climate change, the focus of this study is on no-till and cover crops. In particular, cover crops planted between cash crops take-in additional CO_2_ through photosynthesis and prevent soil erosion, while no-till can avert soil disturbance that would otherwise release CO_2_ back into the atmosphere.

U.S. agricultural lands can provide low-cost opportunities to sequester and reduce emissions of CO_2_ and other GHGs. Lewandrowski et al. [10] estimated that between 1.5 and 36.7 million metric tons of carbon dioxide equivalent^1^ units (mtCO_2_e) could be permanently sequestered in U.S. agricultural lands with a carbon price of $2.73 mtCO_2_e^−1^, afforesting croplands and pasture, shifting cropland to permanent grasses, and increasing the implementation of production practices and rotations that can raise soil-carbon levels (mainly no-till).^2^ A report by the National Academy of Sciences [14] estimated that U.S. agricultural lands could potentially sequester 250 million mtCO_2_e annually via conservation practices to enhance SOC storage, equivalent to around 4% of the country’s emissions. Sperow [24] concluded that the adoption of winter cover crops and no-till can sequester, respectively, 64.9 and 67.5 million mtCO_2_e annually as soil organic carbon under the assumption of full adoption of each practice across the 48 states.^3^ However, Sperow [24] did not evaluate the economic feasibility of full-scale adoption, while cover cropping and no-till practices have only been adopted in 3.9% and 26.4% of croplands in the 48 contiguous states [22], according to the 2017 Census of Agriculture [28]. The low implementation rates of the selected conservation practices along with previous exploratory studies of carbon sequestration in the U.S. agricultural sector suggest that the sector has a large potential to reduce GHG emissions and sequester carbon under the right set of incentives.

The U.S. Department of Agriculture (USDA) offers technical and financial assistance to agricultural producers for the implementation of conservation practices on working lands through the Natural Resources Conservation Service (NRCS).^4^ The largest NRCS program is the Environmental Quality Incentives Program (EQIP), which offers cost-share payments to farmers and ranchers who adopt conservation practices that address at least one of the local resource concerns identified by NRCS: degradation of the soil, water, air, plants, animals, or energy resource base. EQIP offers financial incentives of up to 75% of the NRCS estimated implementation cost for new conservation practices, depending on practices and location [30].

Additionally, U.S. agricultural producers are increasingly able to participate in private voluntary carbon initiatives that compensate their efforts to sequester CO_2_, reduce GHG emissions, and provide ecosystem services through eligible conservation practices [19].^5^ Cover crops and no-till are among the most common eligible practices. All private voluntary carbon initiatives require additionality (i.e., the practice would not have been implemented in the absence of the carbon payment)^6^ and permanence (i.e., the sequestered CO_2_ should not be re-released into the atmosphere for a pre-determined period of time). Private voluntary carbon initiatives measure, monitor, report, and verify (MMRV) the GHG sequestration or emission avoidance resulting from changes in agricultural practices and then sell the resulting carbon credits to private entities looking to offset their own emissions or to reduce emissions from their value chains. The demand for agricultural carbon credits stems from net-zero emissions pledges made by almost one thousand companies worldwide [21]. For example, Carbon by Indigo, a carbon farming initiative by Indigo Ag, created about 130,000 carbon credits in 2022–2023 [8] that were sold to a network of global partner companies, including JPMorgan Chase & Co., Barclays, and The North Face [9].

Some private voluntary carbon initiatives allow participating agricultural producers to receive EQIP cost-share payments and payments for CO_2_ sequestration for the same conservation practice (i.e., “stacking” payments). While EQIP provides technical and financial support to agricultural producers to address local resource concerns through conservation practices, cost-share payments from this program are not directly tied to GHG sequestration or emission avoidance [31]. However, some conservation practices that sequester carbon and are eligible for EQIP are also eligible for private carbon initiatives. The stacking of payments from public programs and private carbon initiatives can provide a stronger economic incentive to farmers than either of them separately.

Despite the environmental benefits stemming from conservation practices (e.g., reduced soil erosion, improved water infiltration, mitigation of nutrient loading in surface waters, and improved soil health), the private costs faced by agricultural producers to implement them is one of the major barriers to large-scale adoption [6, 7, 16, 17, 20]. For example, the annual implementation costs for cover crops and no-till across the United States in 2023 averaged $202.87 and $55.30 ha^−1^, respectively [26]. Furthermore, when private carbon payments are based on the volume of CO_2_ sequestration (“payments per output”), the variability of CO_2_ sequestration potential across soil types, cropping systems, and historical carbon losses [1] affects the compensation to farmers and ranchers across locations. For example, switching from a conventional tillage system in non-irrigated land to a no-till system can annually sequester, on average, as little as 0.15 mtCO_2_e ha^−1^ in La Paz County, Arizona, and as much as 1.85 mtCO_2_e ha^−1^ in Cedar County, Missouri [25]. Moreover, switching tillage practices on irrigated croplands in Cedar County, Missouri, can annually capture 2.57 mtCO_2_e ha^−1^, on average, or 39% more CO_2_ than on non-irrigated land in the same county.

The goal of this study is to examine the potential effects of alternative private payment regimes (per practice versus per output), prices paid to farmers relative to out-of-pocket costs (low vs. high), and granularity of the GHG model (expected county mean versus actual sequestration at the farm level), on the adoption rate of cover crops and no-till in each county of the United States, the resulting CO_2_ sequestration, and farmers’ net returns.

## Methods

Our analysis relies on a highly stylized model of heterogeneous farms [18] calibrated with 2017 U.S. county-level data on conservation practice adoption [22], and simulated with current generalized estimates of GHG impacts of conservation practices from COMET-Planner [25] under alternative carbon payment regimes.

### Theoretical framework

We first describe farmers’ decision-making process in the baseline, in the absence of carbon payments. Then, we introduce two alternative scenarios characterized by different carbon payment regimes, tied to the granularity of information in the underlying GHG model: payment per practice with limited information versus payment per output with full information. In an attempt to focus on the most basic economic problems involved in carbon farming, our model does not address complexities stemming from information asymmetries, strategic behaviors of the economic agents, risks involved in signing multi-year contracts to implement new agricultural practices, or uncertainties surrounding the ex-post quantification of actual amounts of CO_2_e sequestration over the life of the contract, which can hinder the development of agricultural carbon markets [32].

### Baseline scenario: no carbon payments

We assume a unit mass of hectares in each county *j*, with each hectare *i* characterized by ${\theta }_{ji}$, an index of heterogeneity that measures the agronomic appropriateness of a conservation practice in terms of reduced soil erosion, improved water dynamics, yield level and risks, and other agronomic factors. Cropland is assumed to be uniformly distributed within each county with respect to ${\theta }_{ji}$, and can be ordered from the hectare for which the conservation practice is least appropriate and generates no private-benefit (${\theta }_{ji}=0$), to the hectare for which the conservation practice is most appropriate and generates the highest possible level of private-benefit (${\theta }_{ji}=1$): ${\theta }_{ji}\sim {U}_{j}\left[\mathrm{0,1}\right],\forall j$. For tractability, we assume that decisions are made on a per-hectare basis although we recognize that land and machinery indivisibilities generate economies of scale that are not captured by this stylized model. Farmers are assumed to maximize the net returns to a conservation practice by choosing whether to adopt it or not, according to:

1 $${\pi }_{ji}^{B}=\left\{\begin{array}{c}-{C}_{j}+{\lambda }_{j}{\theta }_{ji} \\ 0\end{array}\begin{array}{c}if \,practice \,is \,adopted \\ if \,practice \,is \,not\, adopted\end{array}\right.$$

where $${C}_{j}$$ is the county-specific out-of-pocket cost to implement the conservation practice, in dollars per hectare ($ ha^−1^), after accounting for all available cost-share payments from government-sponsored programs; $${\lambda }_{j}$$ is the county-specific marginal return to the conservation practice; and $${\lambda }_{j}{\theta }_{ji}$$ is the hectare-specific agronomic private benefit from the implementation of the conservation practice, also in $ ha^−1^.

**Fig. 1:**
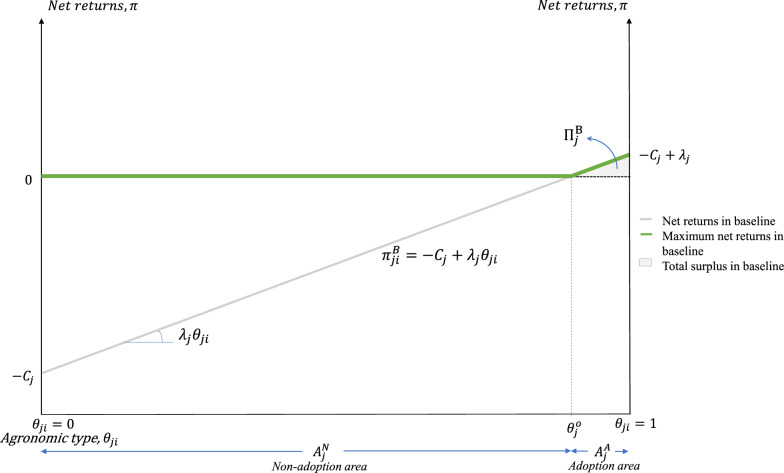
Baseline scenario: no carbon payments

Figure 1 illustrates the baseline scenario for a hypothetical county *j*. Let ${A}_{j}$ be total cropland area in county *j*. The conservation practice is only adopted in those hectares where net returns are non-negative, i.e. all hectares characterized by ${\theta }_{ji}\ge {\theta }_{j}^{o}={C}_{j}/{\lambda }_{j}$. Total adoption of the conservation practice under scrutiny amounts to ${A}_{j}^{A}=\left(1-{\theta }_{j}^{o}\right){A}_{j}$ hectares, while the other ${A}_{j}^{N}={\theta }_{j}^{o}{A}_{j}$ hectares do not adopt the practice. The kinked green line in Fig. 1 is the envelope of the maximum net returns for each hectare in the county, and the shaded area under the envelope measures the scaled total net returns to adopters in the absence of carbon farming payments, as a percent of total county area. The total net returns to adopters in county *j* is ${\Pi }_{j}^{B}={A}_{j}{\int }_{{\theta }_{j}^{o}}^{1}{\pi }_{ji}^{B}d{\theta }_{ji}={A}_{j}{\left(-{C}_{j}+{\lambda }_{j}\right)}^{2}/\left(2{\lambda }_{j}\right)$.

We assume that due to the additionality requirement imposed by most private voluntary carbon initiatives, only hectares in ${A}_{j}^{N}$ (which have not adopted the conservation practice in the baseline scenario, and are characterized by ${\theta }_{ji}<{\theta }_{j}^{o}$) are eligible to participate in carbon initiatives in Scenarios 1 and 2.

### Scenario 1: Carbon payments per practice with limited information

**Fig. 2:**
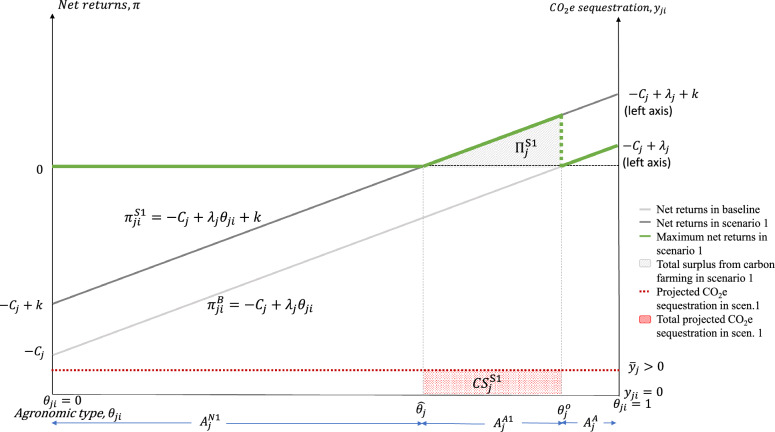
Scenario 1: carbon payments per practice

In this scenario, we assume that carbon initiatives and farmers only know with certainty the average carbon sequestration per hectare per practice at the county level, ${\overline{Y} }_{j}$ mtCO_2_e ha^−1^, but not at the individual hectare level. Consequently, voluntary carbon initiatives offer fixed payments per practice per hectare to all participating farmers in the county, irrespectively of the actual amount of carbon sequestered in each farm. The fixed payment could reflect discounts based on carbon initiatives’ unknown but expected variability of the carbon sequestration across all farms in the county. In this scenario, farmers maximize the net returns to a conservation practice by choosing whether to adopt it, according to:

2 $$\pi _{ji}^{S1} = \left\{ {\begin{array}{*{20}c} { - C_j + \lambda _j \theta _{ji} + k} & {if~\theta _{ji} < \theta _j^o ~\& ~practice~is~adopted~with~carbon~payment} \\ 0 & {if~practice~is~not~adopted~} \\ \end{array} ,} \right.$$

where $$k$$ is the carbon payment^7^ in $ ha^−1^; $$k\le {C}_{j}$$ to reflect that carbon initiatives will not transfer unnecessary rents to farmers; and the other parameters are as defined above. Figure 2 illustrates Scenario 1. The area under the conservation practice increases by $${A}_{j}^{A1}= \left({\theta }_{j}^{o}-{\widehat{\theta }}_{j}\right){A}_{j}={A}_{j}k/{\lambda }_{j}$$ hectares, where $${\widehat{\theta }}_{j}=\left({C}_{j}-k\right)/{\lambda }_{j}$$ characterizes the farm with the lowest appropriateness index that breaks-even with the carbon payment. Total adoption of the conservation practice amounts to $${A}_{j}^{S1}={A}_{j}^{A1}+{A}_{j}^{A}=\left(1-{\widehat{\theta }}_{j}\right){A}_{j}$$ hectares; leaving $${A}_{j}^{N1}={\widehat{\theta }}_{j}{A}_{j}$$ hectares where the practice is not adopted. The kinked green line in Fig. 2 is the envelope of the maximum net returns for each hectare in the county, and the shaded area under the green line measures the scaled total net returns to new adopters in Scenario 1, as percent of county area. Total net returns to new adopters is $${\Pi }_{j}^{S1}={A}_{j}{\int }_{{\widehat{\theta }}_{j}}^{{\theta }_{j}^{o}}{\pi }_{ji}^{S1}d{\theta }_{ji}={A}_{j}{k}^{2}/\left(2{\lambda }_{j}\right)$$.

Total projected additional carbon sequestration due to carbon farming payments (at the time of contract signing) is calculated as ${TCS}_{j}^{S1}={\int }_{{\widehat{\theta }}_{j}}^{{\theta }_{j}^{o}}{\overline{Y} }_{j}d{\theta }_{ji}={\overline{Y} }_{j}k/{\lambda }_{j}$. Let ${\overline{y} }_{j}={\overline{Y} }_{j}/{A}_{j}$ be the average CO_2_e sequestration per hectare scaled by county area, and let ${CS}_{j}^{S1}={\overline{y} }_{j}k/{\lambda }_{j}$ be the total CO_2_e sequestration in the county scaled by county area, so that ${TCS}_{j}^{S1}={A}_{j}{CS}_{j}^{S1}$. Figure 2 illustrates ${CS}_{j}^{S1}$ as the highlighted red area under the dotted red line (measured on the right axis).

### Scenario 2: carbon payments per output with full information

In this scenario, we assume that both carbon initiatives and farmers know with certainty at the time of contract signing the amount of CO_2_e sequestration that would take place in each hectare over the life of the carbon farming contract, $${Y}_{ji}$$ mtCO_2_e ha^−1^. Carbon farming payments per hectare are determined as the product of the price of carbon credits in $ mtCO_2_e^−1^, $$p$$, and $${Y}_{ji}$$. For model consistency, let $${y}_{ji}={Y}_{ji}/{A}_{j}$$ be the amount of CO_2_e sequestration per hectare scaled by county area. In this scenario, farmers maximize the net returns to a conservation practice by choosing whether to adopt it, according to:

3 $${\pi }_{ji}^{S2}=\left\{\begin{array}{cc}-{C}_{j}+{\lambda }_{j}{\theta }_{ji}+p{y}_{ji}& if\, {\theta }_{ji}<{\theta }_{j}^{o} \, \& \,practice\, is\, adopted \,with \,carbon \,payment\\ 0& if \,practice \,is \,not \,adopted \end{array}\right..$$

Given the multiplicity of factors that influence carbon sequestration across farms (including weather effects, timing of practices, and soil properties) and the lack of evidence linking the degree of agricultural appropriateness of conservation practices to the amount of CO_2_e sequestration, we assume that the distribution of ${y}_{ji}$ is orthogonal to the distribution of ${\theta }_{ji}$ (i.e., the agronomic characteristics that determine the appropriateness of a conservation practice for a farm are independent of the amount of CO_2_e sequestration in that farm).^8^

**Fig. 3:**
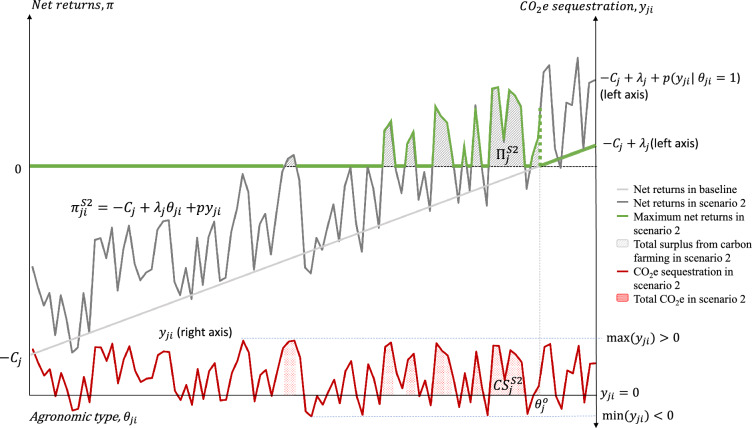
Scenario 2: carbon payments per output

Figure 3 illustrates Scenario 2: ${y}_{ji}$ is graphed as a solid red line at the bottom of the chart measured on the right axis. Most hectares in Fig. 3 sequester carbon, ${y}_{ji}\in (0,{\text{max}}\left({y}_{ji}\right)]$, while a few hectares produce net positive carbon emissions, ${y}_{ji}\in [{\text{min}}\left({y}_{ji}\right),0)$. Since both farmers and carbon initiatives are assumed to know in advance and with certainty the amount of carbon sequestration that will take place over the life of the carbon farming contract, farmers will only enroll hectares for which net returns are non-negative. Due to the assumed independence between ${\theta }_{ji}$ and ${y}_{ji}$, the new adopters can be distributed discontinuously across ${\theta }_{ji}\in \left[0, {\theta }_{j}^{o}\right]$. The actual distribution of ${y}_{ji}$’s must be used to solve the model numerically to determine the number of hectares that will adopt the conservation practice due to carbon payments. The total net returns to new adopters in Scenario 2, ${\Pi }_{j}^{S2}={A}_{j}{\int }_{{\theta }_{ji}:{\pi }_{ji}^{S2}\ge 0}^{{\theta }_{j}^{o}}{\pi }_{ji}^{S2}d{\theta }_{ji}$, equals the sum of the highlighted areas under the green envelope curve, scaled up by county area. Similarly, the total amount of carbon sequestration in this scenario is measured as the sum of areas under the red line at the bottom of Fig. 3, scaled up by county area: ${TCS}_{j}^{S2}={A}_{j}{\int }_{{\theta }_{ji}:{\pi }_{ji}^{S2}\ge 0}^{{\theta }_{j}^{o}}{y}_{ji}d{\theta }_{ji}$.

### Comparing Scenarios 1 and 2

Two major differences between Scenarios 1 and 2 are that in the latter: (1) adoption of the conservation practice need not occur across similar (i.e. contiguous) hectares in the ${\theta }_{ji}$ space, and (2) each carbon payment compensates actual CO_2_e sequestration in every participating hectare, rather than compensating the projected county-average sequestration.

**Fig. 4:**
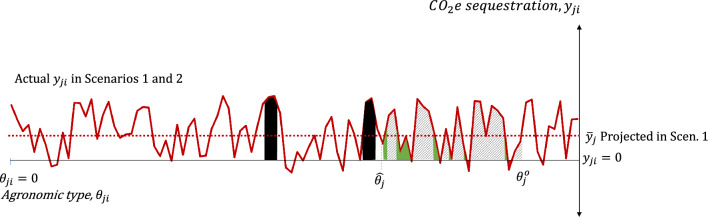
Projected and “Actual” Carbon Sequestration Estimates in Scenarios 1 and 2

Under the assumptions that (a) information on actual CO_2_e sequestration per hectare becomes available to all participating farmers in Scenario 1 at the end of the contract (at no extra cost), and that (b) the actual CO_2_e sequestration per hectare is not affected by the type of payment incentivizing the practice, we can compare (1) projected versus actual carbon sequestration in Scenario 1, and (2) actual carbon sequestration across Scenarios 1 and 2. Figure 4 illustrates the latter comparison: the gray areas indicate carbon sequestration common to both scenarios; the solid green areas, where $0<{y}_{ji}<{\overline{y} }_{j},$ indicate carbon sequestration occurring only in Scenario 1; the solid red areas, where ${y}_{ji}<0$, indicate net carbon emissions that take place only in Scenario 1; finally, the solid black areas indicate positive carbon sequestration occurring only in Scenario 2. Naturally, the difference in carbon sequestration across scenarios can be calculated by subtracting the green areas from the summation of the black and red areas. Figure 4 illustrates a case where total carbon sequestration under Scenario 2 is higher than under Scenario 1.

## Data and baseline calibration

We calibrated the baseline model using county-level data on cover crops and no-till adoption rates and cropland area from Sawadgo and Plastina [22], based on the 2017 Census of Agriculture [28], and state-level cost estimates for 2017. The out-of-pocket costs in 2017 were estimated for each state as the difference between total practice implementation costs per hectare and EQIP payments per hectare. Data on the state-specific 2017 EQIP payments per hectare were obtained through personal communication with USDA staff. The 2017 implementation costs per hectare were estimated by dividing the 2017 EQIP payments per hectare by the cost-share ratio of EQIP payments to practice implementation costs in 2023 [26]. For example, USDA [26] reports the implementation cost of cover crops in Iowa in 2023 at $201.96 ha^−1^, and the EQIP payment at $100.97 ha^−1^, equivalent to a 50% cost-share ratio. Given a 2017 EQIP payment of $102.27 ha^−1^, the $204.55 ha^−1^ implementation cost estimate for cover crops in Iowa in 2017 was obtained by dividing the 2017 EQIP payment by the 50% cost-share ratio from 2023. Finally, the resulting $102.27 ha^−1^ out-of-pocket cost for cover crops in Iowa in 2017 was estimated by subtracting the EQIP payment from the estimated cost of implementation.

**Fig. 5:**
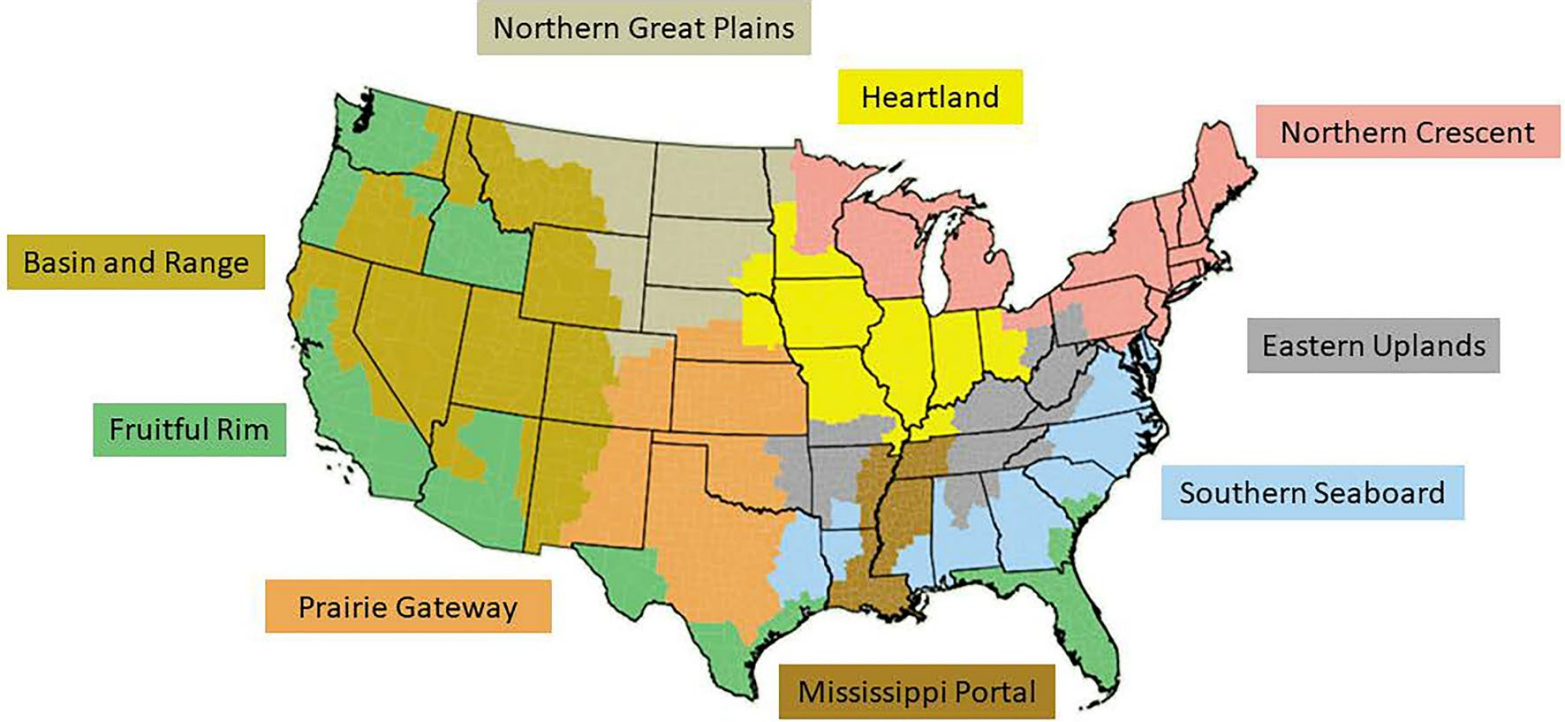
U.S. Farm Resource Regions [29]

Table 1 provides summary statistics for the variables included in the analysis by farm resource region [29]. Each region expands across multiple states (Fig. 5). Adoption rates in the baseline scenario vary substantially across practices, and to a lesser extent across regions within practices. Estimated out-of-pocket costs for cover crops are more than four times the out-of-pocket costs for no-till across all regions, with national averages around $73 and $17 ha^−1^, respectively.

**Table 1:** Descriptive statistics of county-level variables used to calibrate the Baseline

Region	Cover Crops				No-Till			
	2017 Adoption Rate (% of cropland)^*^		Out-of-pocket cost ($ ha^−1^)^^^		2017 Adoption Rate (% of cropland)^*^		Out-of-pocket cost ($ ha^−1^)^^^	
	Mean	StDev	Mean	StDev	Mean	StDev	Mean	StDev
Heartland	4.63	3.55	77.4	27.6	32.53	18.08	18.6	7.81
Northern Crescent	8.13	7.36	56.2	14.1	15.91	14.85	13.7	3.48
Northern Great Plains	1.69	1.25	129	41.2	29.84	22.14	28.1	9.8
Prairie Gateway	2.65	2.69	87.9	20.9	22.03	20.60	21.2	6.42
Eastern Uplands	4.41	4.47	72.2	21.4	14.19	14.95	15.2	4.44
Southern Seaboard	8.63	8.96	55.9	14.3	21.85	20.87	13.3	3.04
Fruitful Rim	4.45	5.84	75.8	25.3	6.69	7.79	17.8	5.87
Basin and Range	2.54	2.85	66.1	18.3	9.35	13.78	15.9	4.75
Mississippi Portal	3.73	4.08	61.6	15.1	21.19	20.53	15.2	5.13
*U.S. Total*	*5.09*	*5.96*	*73.1*	*28.5*	*20.50*	*19.39*	*17.1*	*6.9*

* Source: Sawadgo and Plastina [22]

^^^ Source: authors’ calculations based on USDA (2023a)

We set ${C}_{j}$ equal to the county-specific out-of-pocket cost per hectare for the conservation practice under analysis, and $(1-{\theta }_{j}^{o})$ equal to the 2017 adoption rate, to derive ${\lambda }_{j}={C}_{j}/{\theta }_{j}^{o}$. Annual carbon sequestration at the farm level is simulated using county-specific net changes in GHGs stemming from cover crops and no-till adoption, calibrated with data from COMET-Planner [25] for irrigated and non-irrigated cropland. COMET-Planner is a simulated soil organic carbon (up to a depth of 30.48 cm) and GHG (including carbon dioxide, nitrous oxide, and methane) evaluation dataset for NRCS conservation planning that reports annual changes in net GHG emissions from the adoption of conservation practices, measured in mtCO_2_e per acre, for all counties in the United States.^9^ Several voluntary carbon farming initiatives, including Nori and the Soil and Water Outcomes Fund, use carbon models with similar underlying methods to quantify carbon sequestration [19]. Since the summary statistics from COMET-Planner [25] cannot be consistently approximated across counties by any of the common parametric distributions,^10^ we decided to represent the variability in CO_2_e sequestration across practices and locations through simple triangular distributions. This allowed us to use the reported lower and upper limits to CO_2_e sequestration and impute the reported average CO_2_e sequestration as the most frequent outcome (mode) of our triangular distributions for irrigated and non-irrigated land in each country.^11^ The goal was to reflect the variability in the potential to sequester carbon (including the possibility of producing net carbon emissions) associated with each conservation practice and county, rather than to produce precise estimates of CO_2_e sequestration through voluntary carbon initiatives.

While Swan et al. [25] provide CO_2_e sequestration estimates by irrigation type, adoption rates and out-of-pocket costs of conservation practices are not available at such level of disaggregation. In order to integrate the triangular distributions across counties with irrigation, data on cropland area under irrigation from the 2017 Census of Agriculture [28] were used as weights for the random draws from the irrigated and non-irrigated triangular distributions for each practice. As show in Table 2, cover cropping has a relatively lower carbon sequestration potential compared to no-till and higher potential to generate negative carbon sequestration (positive emissions) across all regions except for the Mississippi Portal. The national annual average carbon sequestration potentials via cover crops and no-till are, respectively, 0.5679 and 1.0197 mtCO_2_e ha^−1^. While the Mississippi Portal is the region with highest mean carbon sequestration potential by via cover crops (equivalent to 2.7 times the national average), the Heartland is the one with the highest mean carbon sequestration potential via no-till (equivalent to 1.3 times the national average).

**Table 2:** Annual carbon sequestration by conservation practice

Region	Cover Crops (mtCO_2_e ha^−1^)			No-Till (mtCO_2_e ha^−1^)		
	Mean	Min	Max	Mean	Min	Max
Heartland	0.6961	− 0.2992	4.6956	1.3556	− 0.2137	3.4011
Northern Crescent	0.2198	− 0.2198	1.9234	1.1174	− 0.2137	2.9615
Northern Great Plains	0.1160	− 0.9587	0.8732	0.6778	− 0.3664	1.9051
Prairie Gateway	0.3603	− 1.0075	3.1752	0.8182	− 0.6289	3.3584
Eastern Uplands	0.8732	− 0.2748	4.7567	1.2395	− 0.0366	3.4744
Southern Seaboard	0.7327	− 0.2442	4.7567	1.0625	− 0.0366	3.3645
Fruitful Rim	0.4641	− 2.4669	4.1522	0.7083	− 1.1724	3.8774
Basin and Range	0.0672	− 2.4669	1.9478	0.3297	− 1.1724	3.2301
Mississippi Portal	1.5204	− 0.3786	4.8971	1.2456	− 0.0244	3.5415
*U.S. Total*	*0.5679*	− *2.4669*	*4.8910*	*1.0197*	− *1.1724*	*3.8774*

Source: authors’ calculations based on Swan et al. [25]

## Simulated results

### Results from Scenario 1

In line with the carbon prices paid by voluntary carbon initiatives reported by Plastina and Wongpiyabovorn [19], we assume $k=\$12.36$ ha^−1^ for both practices across all counties in the nation. Note that such payment represents 17% of the national average out-of-pocket cost for cover crops, but 72% of the corresponding costs for no-till.

For each practice, we set the projected average amount of carbon sequestration per county, $${\overline{Y} }_{j}$$, equal to the irrigation-weighted mean of the underlying triangular distributions of carbon sequestration. Table 3 shows that carbon farming would induce the expansion of cover crop area by 25.76 million hectares across the United States, equivalent to 16% of total cropland. Seventy percent of the extra area in cover crops would come from the Heartland (29%), followed by the Prairie Gateway (18%), the Northern Great Plains (12%), and the Northern Crescent (11%). The net returns to farmers in those four regions would account for 76% of the $787.6 million obtained by all farmers in the nation. However, only two regions from this list would contribute more than 10% of the total national projected sequestration (13.88 million mtCO_2_e): the Heartland and the Prairie Gateway, with 35% and 14%, respectively. The Mississippi Portal and the Sothern Seaboard would contribute, respectively, 18% and 11% of the national total. Interestingly, the Basin and Range would actually generate slightly negative net carbon sequestration, or net positive emissions, due to the very low carbon sequestration potential through cover crops in that region (Table 2).

**Table 3:** Simulation Results from Scenario 1

Regions	2017 Cover Crop Adoption Rate (% of crop-land)	Results from new adoption of Cover Crops due to Carbon Farming				2017 No-Till Adoption Rate (% of crop-land)	Results from new adoption of No-Till due to Carbon Farming			
		Additional Area in Cover Crops		Projected Additional Carbon Sequestration (thousand mtCO_2_e)	Additional Net Returns to Farmers (million US$)		Additional Area in No Till		Projected Additional Carbon Sequestration, (thousand mtCO_2_e)	Additional Net Returns to Farmers (million US$)
		Million ha	% of cropland				Million ha	% of cropland		
Heartland	4.19	7.46	16.25	4,867	261.3	30.69	*22.28*	48.52	26,979	262.2
Northern Crescent	7.84	2.92	20.35	865	78.5	19.46	*10.24*	71.35	10,178	78.7
Northern Great Plains	1.47	3.04	11.12	421	130.3	34.02	*9.36*	34.22	5,903	132.3
Prairie Gateway	2.69	4.7	14.33	1,954	131.7	28.13	*14.65*	44.71	11,628	135.8
Eastern Uplands	4.66	1.18	18.14	1,077	33.7	19.03	*4.48*	68.86	5,771	34.4
Southern Seaboard	11.08	1.49	19.72	1,533	37.7	27.47	*4.88*	64.79	5,633	38.1
Fruitful Rim	3.01	2.38	19.58	754	53.3	6.25	*9.14*	75.28	7,593	51.9
Basin and Range	1.58	1.29	19.72	-22	25.3	17.53	*4.33*	66.17	2,158	27.2
Mississippi Portal	3.50	1.3	18.15	2,433	35.8	21.37	*4.61*	64.44	6,703	37.4
*U.S. Total*	*3.88*	*25.8*	*16.07*	*13,881*	*787.6*	*26.35*	*83.97*	*52.39*	*82,545*	*797.9*

Sources: authors’ calculations, except for 2017 adoption rates [22]

No-till would be adopted in 83.97 million additional hectares across the nation due to carbon farming payments, sequestering 82.55 million mtCO_2_e per year. This very high projected increase in no-till use is mainly driven by the assumption of low out-of-pocket costs in the baseline, and the unrealistic implicit assumption that implementation costs would remain constant after an explosion in demand for no-till equipment. The simulated changes in no-till patterns due to carbon farming incentives should be considered beyond optimistic, and are only reported in this study to highlight the importance of discussing the future of carbon markets in terms of the share of private implementation costs covered by carbon payments rather than solely on the absolute magnitude of the carbon payment.

The Heartland and the Prairie Gateway would account, respectively, for 27% and 17% of the additional area in no-till, and 33% and 17% of the $797.9 million in national net returns to farmers, and 33% and 14% of the carbon sequestration. The Northern Crescent is the third region in order of relevance in carbon sequestration and additional no-till area, but the Northern Great Plains surpasses it in net returns to farmers: $132.3 versus $78.7 million, or 17% vs. 10% of the national total. Additional net returns to farmers would be $2.3 million higher under no-till than under cover cropping practices at the national level, and all regions would experience higher net returns except the Fruitful Rim (where net returns to no-till would be limited by land availability at the point of nearly full adoption in most counties).

### Results from Scenario 2

We assume $p=\$15$ mtCO_2_e^−1^ for carbon sequestered through both practices across all counties in the nation, in line with the lower bound of prices reported by Plastina and Wongpiyabovorn [19]. We simulate the amount of carbon sequestration per practice per hectare, ${Y}_{ji}$, by generating 10,000 random draws from the county- and practice-specific irrigation-weighted triangular distributions. Scenario 2 assumes that agricultural producers and carbon initiatives know ${Y}_{ji}$ with certainty prior to signing a carbon farming contract, so the actual amount of carbon sequestration occurring over the life of the contract is exactly the same as the contracted amount. Results are presented in Table 4. Similar to Scenario 1, the no-till area would increase substantially more than the cover crop area in Scenario 2: 78.82 versus 17.55 million hectares at the national level, respectively. However, total additional area in each conservation practice would be lower in Scenario 2 than in Scenario 1 because the share of private implementation costs covered by carbon payments depends on the intrinsic capacity of cropland to sequester carbon in the former. Additional area in cover crops would only be higher in Scenario 2 than in Scenario 1 for the Mississippi Portal, the Eastern Uplands, and the Southern Seaboard, the three regions with the highest potential to sequester carbon through this practice (Table 2). Additional area in no-till would only be higher in Scenario 2 than in Scenario 1 for the Heartland, the Eastern Uplands, and the Mississippi Portal, where the mean carbon sequestration potential per county exceeds 1.2 mtCO_2_e ha^−1^ (Table 2).

**Table 4:** Simulation Results from Scenario 2

Regions	Results from new adoption of Cover Crops due to Carbon Farming				Results from new adoption of No-Till due to Carbon Farming			
	Additional Area in Cover Crops		Additional Carbon Sequestration (thousand mtCO_2_e)^	Additional Net Returns to Farmers (million US$)	Additional Area in No Till		Additional Carbon Sequestration (thousand mtCO_2_e)^	Additional Net Returns to Farmers (million US$)
	Million ha	% of cropland			Million ha	% of cropland		
Heartland	5.86	12.77	5,279	223.0	24.5	53.37	29,285	481.3
Northern Crescent	1.06	7.35	425	37.0	9.45	65.87	9,123	127.9
Northern Great Plains	0.61	2.23	155	47.0	7.17	26.2	4,294	124.7
Prairie Gateway	2.52	7.7	1,671	107.8	13.5	41.28	8,413	162.1
Eastern Uplands	1.35	20.76	1,394	48.6	4.72	72.54	5,406	76.6
Southern Seaboard	1.84	24.36	2,124	58.7	4.69	62.2	4,661	72.9
Fruitful Rim	1.23	10.16	1,021	50.2	6.98	57.52	5,757	77.8
Basin and Range	0.15	2.2	38	7.9	2.59	39.63	1,235	24.2
Mississippi Portal	2.93	40.93	5,543	96.2	5.18	72.44	6,613	90.5
*U.S. Total*	*17.6*	*10.95*	*17,649*	*676.5*	*78.8*	*49.18*	*74,787*	*1,237.9*

^Projected carbon sequestration is assumed equal to “actual” carbon sequestration in Scenario 2

Source: authors’ calculation

Aggregate additional net returns to farmers in the Mississippi Portal, the Southern Seaboard, and the Eastern Uplands would be higher in Scenario 2 than in Scenario 1 for both conservation practices, while aggregate net returns to farmers in the Northern Great Plains and the Basin and Range would be lower for both practices. At the national level, implementing cover crops under Scenario 2 would generate 14% lower aggregate net returns than under Scenario 1, but implementing no-till would generate 55% higher net returns. As described in the Discussion section below, differences in carbon sequestration and net returns could influence the lobbying efforts by different economic agents across the U.S. territory to promote one payment regime over the other.

### Projected versus actual carbon sequestration in Scenario 1

The carbon sequestration results presented above for Scenario 1 are based on projected county-averages, because Scenario 1 assumes limited information on carbon sequestration at the farm level, justifying the use of payments per practice rather than payments per output. While payments received by farmers in Scenario 1 do not depend on their individual amount of carbon sequestration, there is value for end users of the resulting carbon credits and society as a whole in knowing whether a per-practice payment regime is prone to systematically under- or over-estimate actual carbon sequestration. Under the assumptions that an entity can measure without error the actual amount of total carbon sequestered in each farm, and that such measurement exactly overlaps with the actual sequestration assumed for Scenario 2, we calculate the difference between projected and actual carbon sequestration for each participating hectare in Scenario 1. Table 5 shows that projected carbon sequestration tends to consistently exceed actual carbon sequestration with payments per practice. At the national level, projected sequestration through cover crops and no-till would over-estimate actual carbon sequestration by 2.1 and 14.2 million mtCO_2_e_,_ or 18% and 21%, respectively. To the best of our knowledge, this is the first study to illustrate the magnitude of the regional gaps between actual and projected sequestration induced by a payment regime. In reality, carbon farming initiatives and society as a whole could partially address this important drawback of the per-practice payment regime under limited information, by discounting the price of carbon credits generated in this system by the over-estimation ratio.

**Table 5:** Difference between Projected and “Actual” Carbon Sequestration in Scenario 1

Regions	Additional Carbon Sequestration (in thousand mtCO_2_e) from new adoption of:			
	Cover Crops		No-Till	
	“Actual”	Difference between Projected (Table 3) and “Actual”	“Actual”	Difference between Projected (Table 3) and “Actual”
Heartland	4,473	394	25,039	1,940
Northern Crescent	767	98	8,983	1,195
Northern Great Plains	350	71	4,554	1,349
Prairie Gateway	1,410	544	7,559	4,069
Eastern Uplands	905	172	4,687	1,084
Southern Seaboard	1,252	281	4,590	1,043
Fruitful Rim	614	140	5,932	1,661
Basin and Range	– 19	– 3	1,440	718
Mississippi Portal	2,011	422	5,538	1,165
*U.S. Total*	*11,762*	*2,119*	*68,323*	*14,222*

Source: authors’ calculations

### Comparing actual carbon sequestration across Scenarios 1 and 2

**Fig. 6:**
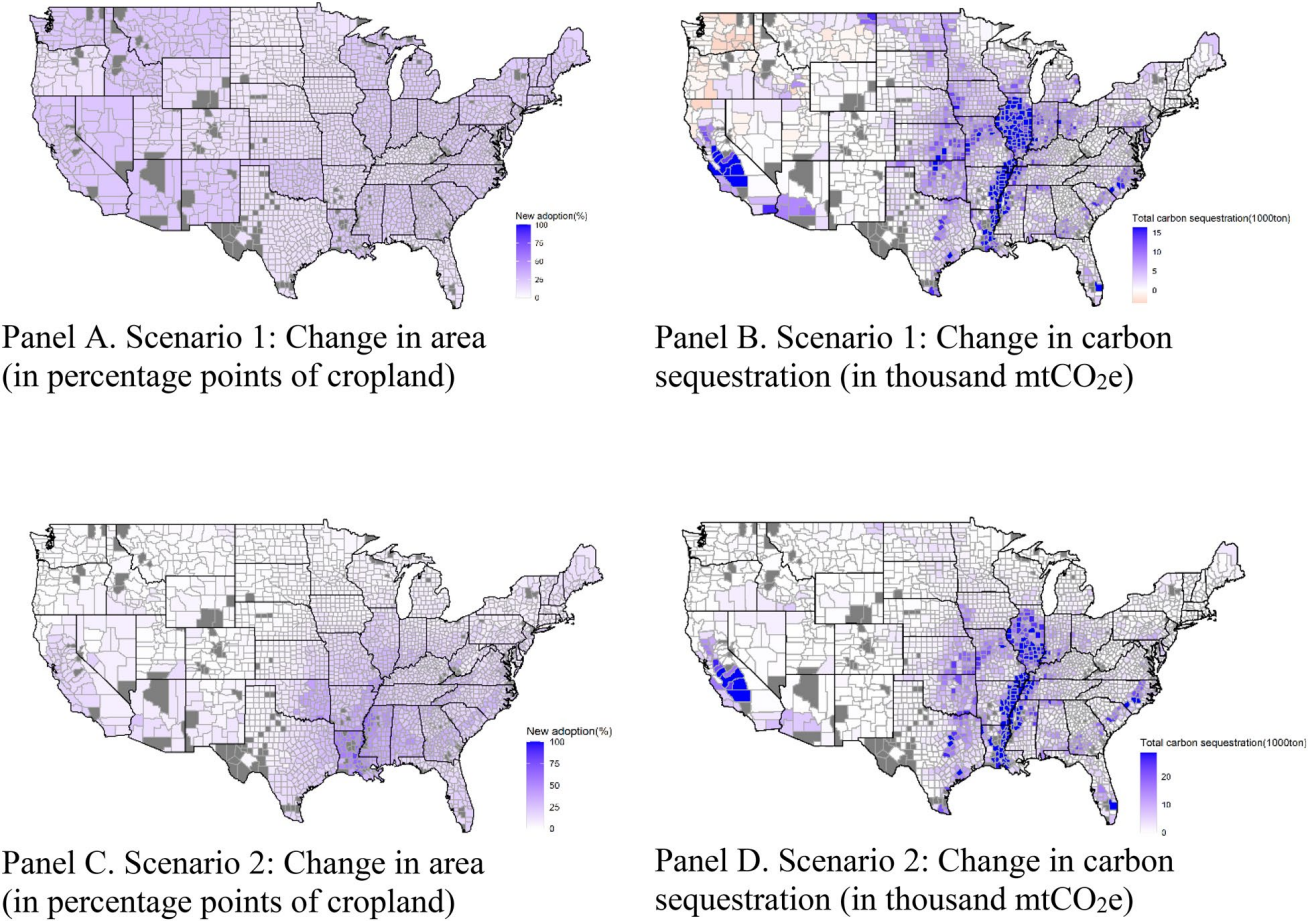
Changes in cover-cropped area and “actual” carbon sequestration by county

Figure 6 illustrates the additional area that farmers would enroll in carbon farming contracts (as a percent of total cropland) and the associated changes in carbon sequestration (in thousand mtCO_2_e) by county under Scenarios 1 and 2. It is evident that payments per practice would incentivize cover crop adoption across the board (Fig. 6 Panel A), even when actual net carbon sequestration is negative for large regions of the country (orange areas in Fig. 6 Panel B), including the Basin and Range, the northern Fruitful Rim, and the Northern Great Planes. A payment per output regime would only enroll areas with positive carbon sequestration (i.e., no orange colors in Fig. 6 Panel D), but losses on the extensive margin (lower adoption rates) across big sections of the Northern Crescent and the Northern Great Plains would offset all gains in the intensive margin (higher carbon sequestration per hectare across participating farms) in those regions. Yet, total actual carbon sequestration via cover crops across the United States would be 5.9 million mtCO_2_e or 50.1% higher in Scenario 2 than in Scenario 1, despite the 5.12% lower adoption rate (14.8% vs. 20.0%). In both scenarios, the counties with highest carbon sequestration would be located around the Mississippi River, in the San Joaquin Valley, the Southern Seaboard, and Palm Beach County in Florida.

**Fig. 7:**
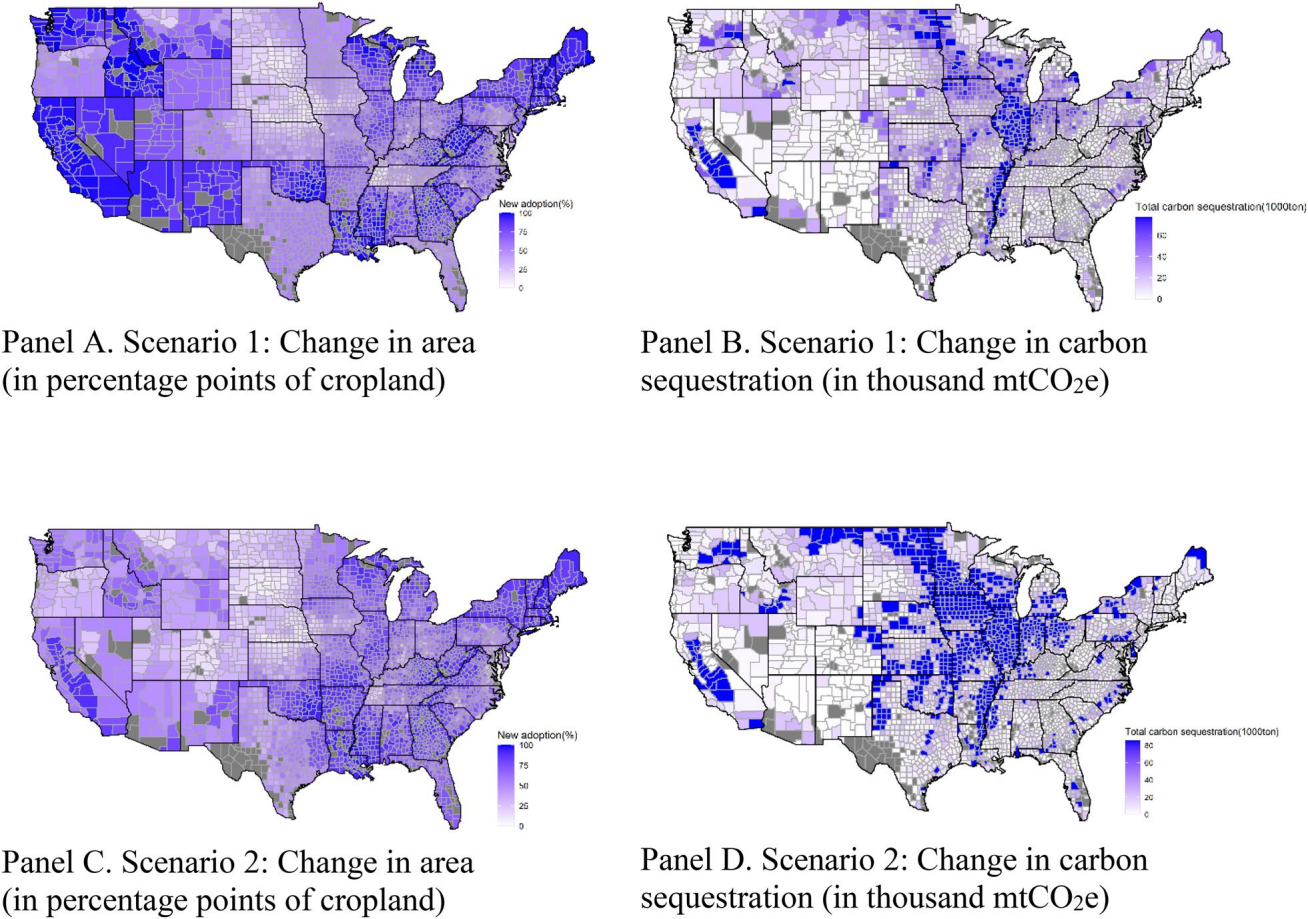
Changes in no-tilled area and “actual” carbon sequestration by county

Panels A and C in Fig. 7 show that a large portion of the country would virtually achieve full adoption of no-till under both Scenarios, with very similar total adoption rates at the national level: 78.74% in Scenario 1 and 75.53% in Scenario 2. Despite the 3.21% lower adoption rate, total actual carbon sequestration in Scenario 2 would be 6.5 million mtCO_2_e or 9.5% higher than in Scenario 1. The Basin and Range, the Northern Great Plains, and the Fruitful Rim would sequester 14%, 6%, and 3% less carbon with 26.54%, 8.20%, and 17.76% lower no-till adoption rates in Scenario 2 than in Scenario 1, respectively. In all other regions, the losses in the extensive margins are more than offset by the gains in the intensive margins.

Two major differences in the patterns of actual carbon sequestration through no-till with respect to cover crops are that no counties produce net positive carbon emissions through no-till (no orange in Fig. 7 Panels B and D); and that the intensity of carbon sequestration through no-till is much higher in the Heartland and the Northern Plains than in other regions.

## Discussion

Carbon farming through cover crops and no-till can be profitable for U.S. farmers: with a carbon price of $15 mtCO_2_e^−1^, private net returns would amount to $676 and $1,234 million, respectively; alternatively, with a carbon payment of $12.36 ha^−1^, private net returns would amount to $788 and 798 million, respectively. However, even the highest estimate only represents 1.2% of the 2017 net cash farm income in the United States, and less than 1% of annual average net cash farm income over 2018–2023 [27]. Consequently, carbon farming cannot be expected to become a major stand-alone priority for U.S. policy makers unless carbon prices increased substantially or net private costs to implement the selected conservation practices dropped dramatically. Additionally, since our model assumes that all farmers participating in carbon farming receive EQIP cost-share payments, the expansion of cover cropped and no-till area would require, respectively, increased federal funding at an annual rate of 3.38 and 2.75 billion dollars in Scenario 1; and 2.37 and 2.55 billion dollars in Scenario 2. The extra EQIP allocations for both practices in Scenarios 1 and 2 represent, respectively, 162% and 130% of the 2023 EQIP budget, including the additional funding from the Inflation Reduction Act of 2022 [13].

Our very optimistic results suggest that the U.S. agricultural sector could sequester between 11.8 and 74.8 million mtCO_2_e annually via cover crops and no-till, respectively. These figures are only a fraction of the potential 250 million mtCO_2_e reported by the National Academy of Sciences [14], suggesting that failing to incorporate economic variables and agronomic differences across counties into the analysis could result in tremendous overestimation of the likely sequestration. Furthermore, our estimates represent between 0.7 and 3.0% of the minimum annual target sequestration of 2.49 billion mtCO_2_e (0.68 billion mt of carbon)^12^ necessary to reduce global temperature by 0.1 °C by 2100 under an aggressive emissions reduction scenario [12].

The extra actual carbon sequestered in Scenario 2 compared to Scenario 1 at the national level through both no-till and cover crop practices underscores the higher effectiveness to generate carbon credits of a per-outcome payment regime than a per-practice payment regime. In addition, while the carbon price is fixed at $15 mtCO_2_e^−1^ in Scenario 2, the outcome-equivalent average prices from Scenario 1 (obtained by multiplying the additional hectares in carbon farming by $5 ha^−1^ and dividing by total U.S. extra carbon sequestration) amount to $27.06 mtCO_2_e^−1^ for cover crops and $15.19 per mtCO_2_e^−1^ for no-till. Payments per practice tend to incentivize adoption of conservation practices over larger areas than payments per outcome, but areas with positive carbon emissions are allowed to participate, reducing the effectiveness of voluntary carbon initiatives under payments per practice.

No pricing regime consistently generates higher aggregate net returns to farmers across all U.S. regions: payments per output would be preferred by farmers adopting no-till in all regions but the Northern Great Plains and the Basin and Range; but payments per practice would be preferred by farmers adopting cover crops in all regions but the Eastern Uplands, the Southern Seaboard, and the Mississippi Portal.

Differences in net returns and carbon sequestration across payment regimes and conservation practices are expected to influence the political economy of agricultural carbon markets: based on aggregate net returns, farmers could be expected to prefer carbon payments per practice for cover crops and payments per output for no-till; and users of carbon credits and society in general could be expected to prefer carbon payments per output for cover crops and payments per practice for no-till.^13^ However, carbon farming initiatives that voluntarily contract with farmers to generate carbon credits from agricultural practices will follow individual pricing and contractual strategies to achieve their own private business interests.

Whereas this analysis presents an approach to integrate the economic, agronomic, and GHG dynamics of agricultural carbon markets in the United States, it lacks granularity on the carbon dynamics, relies on strong economic assumptions, and ignores second-order effects (such as the major increase in machinery costs and initial yield declines for unexperienced farmers with large scale adoption of no-till practices, or the major increase expected in cover crop seed prices following a substantial expansion in demand and the multi-year process necessary to reproduce seeds). Furthermore, the out-of-pocket costs used in our simulations exclude farmers’ opportunity costs from learning how to effectively implement conservation practices, yield effects on the following cash crop, and any co-benefits from agricultural conservation practices. For example, fields in continuous no-till could experience declining operating costs over time and higher land values [3, 4], and grazing or harvesting cover crops as livestock feed could result in substantially lower net costs to agricultural producers [11, 15].

In this study, carbon farming initiatives were assumed to operate under one pricing regime or the other, and to offer a unique price to all farmers in the nation, while recent surveys (e.g. [19]) indicate that there are slightly less than two-dozen active carbon initiatives in the United States, investing resources to differentiate themselves from the rest by offering menus of options to farmers and ranchers, and limiting their operations to specific geographical regions and crop rotations. Carbon initiatives follow different MMRV protocols to issue carbon credits without mandated oversight, and operate with different costs structures that allow them to face a segmented market.

A more refined modeling effort would include iterative dynamic interactions between agricultural producers and carbon initiatives in less than perfectly competitive markets, where producers would have less information about carbon market dynamics than managers of the carbon initiatives (asymmetric information), and permanence would be accounted for and remunerated.

## Conclusions

While carbon markets have been in existence for many decades, agricultural producers in the Unites States have recently entertained a growing number of invitations from voluntary private carbon initiatives to sign multi-year contracts to implement conservation practices in exchange for monetary compensation, tied directly or indirectly to the carbon credits generated through those practices. Most initiatives financially compensate producers through payments per output, while some offer payments per practice [19]. The goal of this article was to evaluate the potential extent of the U.S. voluntary agricultural carbon market, the amount of carbon sequestration, and the net returns to farmers under the alternative payment regimes, using a highly stylized economic model of heterogeneous farms calibrated with county-level data for no-till and cover cropping practices.

Our simulated results indicate that agricultural carbon markets can be profitable for U.S. farmers (although with payments at $12.36 ha^−1^ or $15 mtCO_2_e^−1^ they pale in comparison to overall profits from crop and livestock production) and that annual carbon sequestration could range between 17 and 75 million mtCO_2_e. Payments per output were found to incentivize higher carbon sequestration than payments per practice, but the former regime would be less favored by farmers as a unified group than the latter. However, if operators of farms with high carbon sequestration potential could decide the payment regime to be implemented, they would choose the payment per output regime, due to higher net returns per enrolled hectare. To the best of our knowledge, this is the first study to illustrate the magnitude of the gap between actual and projected sequestration induced by a payment regime in voluntary agricultural carbon markets.

While our model addresses additionality, it provides no insights on the permanence of carbon sequestration, and the quality of carbon credits depends on both attributes.

Despite our tight focus on the economic incentives to sequester GHGs through conservation practices, net returns are not always the main driver of farmers’ decision to adopt them, neither GHG sequestration and emission avoidance are the only environmental services provided by farmers through agricultural conservation practices to society. Future research should focus on relaxing the stringent assumptions imposed in our economic model, improve the granularity of information from the GHG model and explicitly account for asymmetric information and dynamic strategic interactions among economic agents, the risk of non-permanence, and the broader private and public costs and benefits of the agricultural conservation practices under consideration.

## Data Availability

Comet-Planner full dataset publicly available repository: pln-50-ui-010109-dot-comet-201514.appspot.com/download. 2017 adoption rates of cover crops and no-till by county: available from the authors upon request. 2017 out-of-pocket costs to implement cover crops and no-till per acre by state: available from the authors upon request.

## Footnotes

1 All GHGs can be expressed in CO_2_e units, according to their relative global warming potential. For example, methane and nitrous oxide have 28 and 273 times the global warming potential of carbon dioxide for a 100-year timescale, respectively [23].

2 The unit of measurement in Lewandrowski et al. [10] was metric ton of carbon, which we converted into CO_2_e units using the conversion rate 1 mt of carbon = 44/12 × 1 mtCO_2_e [2].

3 The unit of measurement in Sperow [24] was metric ton of carbon, which we converted into CO_2_e units using the conversion rate 1 mt of carbon = 44/12 × 1 mtCO_2_e [2]. The full-adoption assumption in Sperow [24] implies that highly erodible land and current land in the Conservation Reserve Program (CRP) would be completely set-aside. Furthermore, winter cover crops would not be used in rotations with hay and in dry climate regions.

4 The USDA Farm Service Agency manages the largest conservation program for non-working lands, the Conservation Reserve Program (CRP).

5 Regulated companies participating in one of the five emissions trading systems (ETSs) for the energy, industrial, and transportation sectors in the United States [34] can meet their mandates by using carbon offsets generated by emission reductions from unregulated sectors. Methane capture from livestock and rice cultivation are activities allowed to produce carbon offsets for some ETSs, but agricultural carbon offsets account for a very small share of the trade [33].

6 A few initiatives allow participation of recent adopters of eligible conservation practices (typically within a five-year window), and offer producers a sign-up bonus in recognition of the recently sequestered CO_2_.

7 Note that $$k$$ could be conceptualized as the product of an average price premium for a low-carbon intensity commodity and the average yield of that commodity. For example, if the price premium for low-carbon intensity corn is $0.98 per metric ton of corn and the average corn yield is 12.55 metric tons ha^−1^, then the carbon payment amounts to $12.30 ha^−1^.

8 This assumption can easily be modified to reflect all kinds of correlations between ${y}_{ji}$ and ${\theta }_{ji}$, by simply expressing ${y}_{ji}={\rho }_{j}{\theta }_{ji}$, where ${\rho }_{j}$ is the linear correlation coefficient between the two variables.

9 Cover crop planting and no-till are codified by NRCS as practices CPS340 and CPS329, respectively.

10 We tried fitting unimodal, bell-shaped distributions in the exponential family and their truncated versions to replicate the COMET-Planner summary statistics to no avail.

11 Using the reported COMET-Planner means as the means of the possible results while assigning the average value to the most frequent outcome in our triangular distributions would violate the parametric relations imposed by the triangular distribution in a large share of the counties.

12 We use a conversion factor of 1 mt of carbon = 44/12 × 1 mt CO_2_e (Brander, 2017).

13 In the hypothetical case that society had to choose only one carbon payment regime for all agricultural practices, and the lobbying capacity of agricultural producers operating farms with high carbon sequestration potential was higher than that of producers operating farms with medium and low carbon sequestration potential, then a payment per outcome regime would receive stronger support than a payment per practice regime: net returns per participating cover cropped and no-tilled hectare average, respectively, $38.55 and $15.71 at the national level in Scenario 2, compared to $30.58 and $9.50, respectively, in Scenario 1.

## References

[1] Amelung W, Bossio D, de Vries W, Kogel-Knabner I, Lehmann J, Amundson R, Bol R, Collins C, Lal R, Leifeld J, Minasny B, Pan G, Paustian K, Rumpel C, Sanderman J, van Groenigen JW, Mooney S, van Wesemael B, Wander M, Chabbi A (2020). Towards a global-scale soil climate mitigation strategy. Nat Commun.

[2] Brander M (2012) Greenhouse gases, CO2, CO2e, and Carbon: What do all these terms mean? Ecometrica. 2023. https://www.ecometrica.com/assets/GHGs-CO2-CO2e-and-Carbon-What-Do-These-Mean-v2.1.pdf.

[3] Che Y, Rejesus RM, Cavigelli MA, White KE, Aglasan S, Knight LG, Dell C, Hollinger D, Lane ED (2023). Long-Term Economic Impacts of No-Till Adoption. Soil Security.

[4] Chen L, Rejesus RM, Aglasan S, Hagen S, Salas W (2023). The Impact of No-Till on Agricultural Land Values in the United States Midwest. Am J Agr Econ.

[5] Food and Agriculture Organization (FAO). 2020. Emissions due to agriculture. Global, regional and country trends 2000–2018. FAOSTAT Analytical Brief Series No 18. Rome. https://www.fao.org/3/cb3808en/cb3808en.pdf.

[6] Gramig BM, Widmar NJO (2018). Farmer preferences for agricultural soil carbon sequestration schemes. Appl Econ Perspect Policy.

[7] Greiner R, Gregg D (2011). Farmers’ intrinsic motivations, barriers to adoption of conservation practices and effectiveness of policy instruments: Empirical evidence from northern Australia. Land Use Policy.

[8] Indigo Ag (2023a) Catalyze Agriculture as a Climate Change Solution. https://www.indigoag.com/carbon/for-corporations Last accessed 5/12/2023

[9] Indigo Ag (2023b) Indigo Ag’ Second Crop of Soil Carbon Credits Grows 5X Validating Agriculture as a Meaningful Solution to Climate Change. PR Newswire. https://www.rb.gy/4lwu88.

[10] Lewandrowski J, Peters M, Jones C, House R, Sperow M, Eve M, Paustian K (2004) Economics of Sequestering Carbon in the U.S. Agricultural Sector. Technical Bulletin Number 1909, Economic Research Service, U.S. Department of Agriculture. https://www.ers.usda.gov/publications/pub-details/?pubid=47481.

[11] Malone RW, O'Brien PL, Herbstritt S, Emmett BD, Karlen DL, Kaspar TC, Kohler K, Radke A, Lence SH, Wu H, Richard TL (2022). Rye–soybean Double-crop: Planting Method and N Fertilization Effects in the North Central US. Renewable Agric Food Syst.

[12] Mayer A, Hausfater Z, Jones AD, Silver WL (2018). The potential of agricultural land management to contribute to lower global surface temperatures. Sci Adv.

[13] Myers S (2022) What’s in the Inflation Reduction Act for Agriculture? American Farm Bureau Federation. https://www.fb.org/market-intel/whats-in-the-inflation-reduction-act-for-agriculture.

[14] Academies N, of Sciences, Engineering, and Medicine. (2019). Negative Emissions Technologies and Reliable Sequestration: A Research Agenda.

[15] Plastina A, Acharya J, Marcos F, Parvej M, Licht M, Robertson A (2023). Does grazing winter cereal rye in Iowa, USA, make it profitable?. Renewable Agric Food Syst.

[16] Plastina A, Liu F, Sawadgo W, Miguez FE, Carlson S, Marcillo G (2018). Annual Net Returns to Cover Crops in Iowa. J Appl Farm Econ.

[17] Plastina A, Liu F, Miguez F, Carlson S (2020). Cover Crops Use in Midwestern US Agriculture: Perceived Benefits and Net Returns. Renewable Agric Food Syst.

[18] Plastina A, Giannakas K, Pick D (2011). Market and Welfare Effects of Mandatory Country-of-Origin Labeling in the American Agri-food System. South Econ J.

[19] Plastina A, Wongpiyabovorn O. How to Grow and Sell Carbon Credits in US Agriculture. Ag Decision Maker File A1–76. Iowa State University Extension and Outreach. 2023. https://www.extension.iastate.edu/agdm/crops/pdf/a1-76.pdf. First Published July 2021.

[20] Ranjan P, Wardropper CB, Eanes FR, Redday S, Harden SC, Masuda YJ, Prokopy LS (2019). Understanding barriers and opportunities for adoption of conservation practices on rented farmland in the US. Land Use Policy.

[21] Sanderson K (2023). Net-zero pledges are growing – how serious are they?. Nature.

[22] Sawadgo W, Plastina A. The Invisible Elephant: Disadoption of Conservation Practices in the United States. Choices Magazine. Volume 37. Quarter 1. 2022.

[23] Smith C, Nicholls ZRJ, Armour K, Collins W, Forster P, Meinshausen M, Palmer MD, Watanabe M. The earth’s energy budget, climate feedbacks, and climate sensitivity supplementary material. In Climate change 2021: The physical science basis. Contribution of Working Group I to the Sixth Assessment Report of the Intergovernmental Panel on Climate Change [Masson-Delmotte, V., P. Zhai, A. Pirani, S.L. Connors, C. Péan, S. Berger, N. Caud, Y. Chen, L. Goldfarb, M.I. Gomis, M. Huang, K. Leitzell, E. Lonnoy, J.B.R. Matthews, T.K. Maycock, T. Waterfield, O. Yelekçi, R. Yu, and B. Zhou (eds.)]. 2021. https://www.ipcc.ch/report/ar6/wg1/downloads/report/IPCC_AR6_WGI_Chapter07_SM.pdf

[24] Sperow M (2020). Updated Potential Soil Carbon Sequestration Rates on U.S. Agricultural Land Based on the 2019 IPCC Guidelines. Soil Tillage Res.

[25] Swan A, Easter M, Chambers A, Brown K, Williams SA, Creque J, Wick J, Paustian K. COMET-Planner Dataset, Version 3.0, Build 1, and COMET-Planner Report: Carbon and Greenhouse Gas Evaluation for NRCS Conservation Practice Planning. A companion report to https://www.comet-planner.com. 2022.

[26] U.S. Department of Agriculture. Payment Schedules (Rates) by State. Natural Resources Conservation Service. 2023a. https://www.nrcs.usda.gov/getting-assistance/payment-schedules.

[27] USDA. Data Files: U.S. and State-Level Farm Income and Wealth Statistics. Washington, DC: U.S. Department of Agriculture, Economic Research Service. 2023b. https://www.ers.usda.gov/data-products/farm-income-and-wealth-statistics.

[28] USDA. 2017 Census of Agriculture. Washington, DC: U.S. Department of Agriculture, National Agricultural Statistical Service. 2019.

[29] USDA. Farm Resource Regions. Washington, DC: U.S. Department of Agriculture, Economic Research Service, Agricultural Information Bulletin AIB-760, September. 2000.

[30] Wongpiyabovorn O, Plastina A. Financial Support for Conservation Practices: EQIP and CSP. Ag Decision Maker File A1–39. Iowa State University Extension and Outreach. 2023a. https://www.extension.iastate.edu/agdm/crops/html/a1-39.html

[31] Wongpiyabovorn, O. and A. Plastina. Carbon Farming: Stacking Payments from Private Initiatives and Federal Programs. Ag Decision Maker File A1–40. Iowa State University Extension and Outreach. 2023b. https://www.extension.iastate.edu/agdm/crops/html/a1-40.html

[32] Wongpiyabovorn O, Plastina A, Crespi JM (2023). Challenges to voluntary Ag carbon markets. Appl Econ Perspect Policy.

[33] Wongpiyabovorn, O., A. Plastina, J.M. Crespi. Policies to Reduce GHG Emissions Should Look to Agricultural Carbon Markets. ProMarket, Chicago Booth Stigler Center for the Study of the Economy and the State. March 6. 2023b.

[34] World Bank. 2023. Carbon Pricing Dashboard. https://www.carbonpricingdashboard.worldbank.org/ Last accessed 05/12/2023.

[35] Zomer RJ, Bossio DA, Sommer R, Verchot LV (2017). Global sequestration potential of increased organic carbon in cropland soils. Sci Rep.

